# Metagenomic Insights into Coal Slag Remediation Effects on Soil and Microbial Health in Qinghai’s Muli Coal Mine

**DOI:** 10.3390/microorganisms12112222

**Published:** 2024-11-01

**Authors:** Qi Lin, Pan Yang, Yongbei Zhang, Wenfei Zhang, Hongping Wu

**Affiliations:** 1 Ministry of Education Key Laboratory for Ecology of Tropical Islands, College of Life Sciences, Hainan Normal University, Haikou 571158, China; 20202071300170@hainnu.edu.cn (Q.L.); y486269195@gmail.com (P.Y.); 2 Hainan Beiou Bio-Energy Development Co., Ltd., Haikou 570100, China; zybei2013@yeah.net

**Keywords:** coal mining, high-altitude regions, soil quality, microbial community, ecological restoration

## Abstract

Long-term coal mining in the Muli coal mine area of Qinghai Province has degraded soil quality and reduced microbial diversity, making it imperative to implement effective ecological restoration measures to restore soil quality and enhance ecosystem functions. This study evaluated soil samples under 11 ecological restoration treatments using metagenomic sequencing combined with soil quality analysis to explore the responses of the microbial community structure and function to identify effective restoration measures. This study demonstrated that ecological restoration significantly increased the soil microbial diversity and richness, with the MLII1 (soil samples treated with a chemical weathering agent, attapulgite, and a microbial agent) and MLIII1 (soil samples treated with sheep manure (2.4 kg/m^2^), granular organic fertilizer (1.2 kg/m^2^), and the microbial agent) treatment groups performing exceptionally well. Further analysis of the functional networks revealed that although the MLII2 (soil samples treated with the chemical weathering agent and attapulgite) treatment group did not exhibit the highest species diversity, it exhibited the highest functional network complexity. The results of hierarchical clustering analysis showed that the microbial community of the MLII2 treatment group was most similar to that of the natural meadows compared to the other treatment groups. From the perspective of overall ecological restoration, this study concluded that the MLII2 treatment group exhibited the most favorable ecological restoration outcomes. This finding emphasizes the importance of not only enhancing microbial diversity but also prioritizing the restoration of community functions, especially for the recovery of fragile high-altitude ecosystems.

## 1. Introduction

Coal is a widely used fossil fuel that has long been a cornerstone of global energy supply [1]. The extraction and utilization of coal have spanned centuries; however, the accompanying environmental challenges cannot be overlooked. The improper management of coal mining can contaminate air, water, and soil, which poses hazards to human health and the environment [2,3]. Therefore, it is of paramount importance to address these issues, as the long-term effects of coal pollution extend beyond the immediate vicinity of mining areas and affect both regional and global ecosystems [4].

Coal mining disrupts ecosystems in various ways. Local ecological processes are altered due to surface excavation, fissuring, and waste disposal, resulting in changes to the soil’s physical and chemical properties, seed bank, and microbial communities [5,6]. Subsidence, a significant phenomenon associated with underground mining, causes soil stretching, compression, and fracturing, leading to disruption of the cycling of nutrients, alterations in the pH of the soil, the loss of organic matter, reduced nitrogen, phosphorus, and potassium content, and alterations in the bioavailability of toxic elements, which can have a cascading effect on the local environment [7,8]. This degradation threatens not only land productivity but also the biodiversity and ecological balance of the region [9].

Research indicates that soil pollution, caused by coal mining, affects not only the soil itself but also plants and microbial communities, which are integral to the functioning of terrestrial ecosystems [10]. As primary producers, plants are directly influenced by soil quality. The uptake of contaminants can result in physiological stress, stunted growth, and plant death, thereby affecting the entire food chain [11]. Microorganisms are the most active components in the soil environment, playing a crucial role in organic matter decomposition and nutrient cycling [12,13,14]. Soil microbes are highly sensitive to external changes, making them valuable indicators of land degradation and ecological restoration [15,16]. Mining-induced subsidence alters the soil environment, negatively impacting bacterial adaptability, resulting in reduced bacterial abundance and activity. Even after 20 years of land reclamation in coal mining areas, the number of soil bacteria remains lower than in undisturbed regions, although shrub cover plays a crucial role in ecological recovery [10]. Changes in microbial diversity resulting from coal mining can disrupt ecosystem processes, leading to declines in ecosystem services such as nutrient cycling, soil formation, and air and water purification. Such disruptions can have profound effects on ecosystem stability and resilience, potentially causing long-term changes in the microbial community structure and function [17]. Consequently, it is of paramount importance to understand the intricate interrelationships between coal mining, soil contamination, and their influence on plants and microbial communities. This understanding is essential for the development of effective strategies to mitigate the environmental impacts of coal mining and facilitate the restoration of affected ecosystems [11].

In China, Shanxi, Shaanxi, and Inner Mongolia are the primary focus areas for coal mining research due to their substantial coal reserves and extensive mining activities. However, with economic development, resource exploitation on the Qinghai–Tibet Plateau has also intensified [18]. These conditions, including harsh climates and low biodiversity, exacerbate the environmental challenges associated with coal mining, rendering these areas particularly susceptible to disturbances and significantly impeding ecological recovery [19,20]. Plateau soils, which serve as crucial buffers and support systems for terrestrial ecosystems, are particularly vulnerable to the impacts of coal mining activities [17]. For example, the Muli coal mine, the largest coal mine on the Qinghai–Tibet Plateau, is surrounded by alpine meadows, marshes, and permafrost, which have garnered significant attention in recent years due to ecological issues, such as permafrost degradation [18]. However, there is limited research on the impact of Muli coal mining on ecosystems in high-altitude regions. Therefore, a more comprehensive understanding of the long-term and cumulative effects of coal mining pollution on high-altitude and cold-region ecosystems is required to determine the most effective restoration practices for affected areas and facilitate ecosystem recovery.

Based on the aforementioned research gap, this study focused on the Muli coal mine in Qinghai Province, a region severely affected by coal mining activities, and aimed to evaluate the effectiveness of 11 coal slag treatment measures for improving soil health and enhancing microbial diversity. To accomplish these objectives, we employed metagenomic sequencing to investigate the composition of the soil microbial community, along with analyses of the soil physicochemical properties. It was postulated that integrating metagenomic analysis with soil characteristics would reveal significant differences in microbial diversity and community composition among different treatment strategies and identify the optimal restoration approach. It is anticipated that the most effective remediation measures will be characterized by more diverse and functionally rich microbial communities and improved soil physicochemical properties, indicating enhanced ecosystem resilience and fertility.

## 2. Materials and Methods

### 2.1. Field Trial and Treatments

The Muli coal mine in Tianjun County, Qinghai Province, China (37.2° N, 101.5° E), at an average altitude of 4000 m exemplifies a typical cold high-altitude climate. The annual average temperature is approximately −5.3 °C, with summer highs reaching 15.6 °C and winter lows plummeting to −17.2 °C. The annual precipitation in this region ranges between 400 and 500 mm, with the majority of the precipitation occurring during the summer months. The soil in this mining area is predominantly alpine and marshy meadow soil, with most of it being permafrost and exhibiting widespread permafrost layers at varying depths [21,22]. Coal mining operations at the Muli coal mine have ceased, and comprehensive ecological restoration projects, which began in July 2022, are underway. Soil samples were collected in July 2023 from an area designated for ecological restoration within a coalfield. This area encompasses 12 types of soil, with detailed information available in Table S1.

### 2.2. Sample Collection and DNA Extraction

A total of 36 samples were collected, each with three biological replicates for the 12 types of soil. Each sample comprised 100 g of surface non-rhizosphere soil (5–15 cm) from five subsamples, which were mixed to form a single biological sample. All soil samples were transported on dry ice to the laboratory and stored at −80 °C until DNA extraction. Soil characteristics, including water content (WC), organic matter (OM), total nitrogen (TN), total phosphorus (TP), total potassium (TK), available nitrogen (AN), available phosphorus (AP), available potassium (AK), hydrogen (pH), microbial biomass carbon (MBC), microbial biomass nitrogen (MBN), urease (URE), acid phosphatase (ACP), invertase (SUC), and catalase (CAT), were evaluated. A comprehensive assessment of soil quality was conducted based on these indicators.

**Figure 1:**
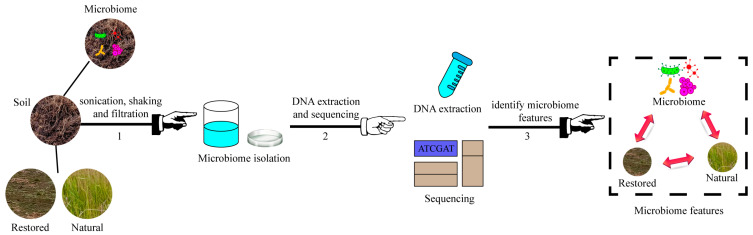
Microbiome feature identification in natural and ecologically restored soils. Workflow for the separation, DNA extraction, and sequencing of soil microbiomes.

Genomic DNA was extracted from 0.4 g soil samples using the PowerSoil DNA Isolation Kit (MO BIO Laboratories, Carlsbad, CA, USA). DNA integrity was evaluated using electrophoresis on 1.0% agarose gel, and the concentrations were quantified using a NanoDrop 1000 spectrophotometer (Thermo Scientific, Waltham, MA, USA). Twelve sample sets were prepared, each encompassing soil microbial communities from both the control and treatment cohorts. A total of 36 biological specimens were processed for each group, with the samples designated for subsequent shotgun metagenomic analyses (Figure 1).

### 2.3. Shotgun Metagenomic Sequencing, Assembly, and Annotation

For shotgun metagenomic sequencing, the DNA samples were subjected to sonication, resulting in fragments with an average length of 350 bp. These fragments served as the basis for constructing the sequencing library, which was performed using the NEBNext Ultra DNA Library Prep Kit (Illumina Systems, San Diego, CA, USA). Sequencing was conducted using an Illumina NovaSeq 6000 platform (Illumina Inc., San Diego, CA, USA).

The shotgun metagenomic raw data were subjected to quality enhancement via a sliding window trimming method using Trimmomatic [23], resulting in quality control (QC) reads. Following the filtering process, we excluded concordantly aligned reads to retain only high-quality sequences. The refined reads were then aligned with the National Center for Biotechnology Information (NCBI) non-redundant (NR) database to derive the microbial sequences and their taxonomic classifications. Non-alignable reads of bacteria and fungi were excluded from the subsequent analysis. Microbial sequences from the samples were combined for de novo assembly using MEGAHIT [24]. For the assembled contigs, we predicted open reading frames (ORFs) using Prodigal [25] in metagenomic mode, and constructed NR gene catalogs using CD-HIT [26] with a 95% identity threshold. Metagenomic techniques were employed to analyze the microbial communities within the samples, resulting in the acquisition of 1,587,167,466 sequences. After rigorous QC filtering, 1,518,468,654 high-quality sequences were retained, with 539,727,640 high-quality sequences matching bacteria and fungi, respectively (Table S2).

Gene annotation was performed using the DIAMOND algorithm [27] against the NR database. Functional annotations were performed using the Kyoto Encyclopedia of Genes and Genomes (KEGG) database [28] and the Clusters of Orthologous Groups (COG) database [29]. The Chao1 index of functional diversity was calculated using the rarefied table in the Quantitative Insights Into Microbial Ecology (QIIME).

### 2.4. Statistical Analysis

A principal coordinate analysis (PCoA) [30] and plotting were performed using the ADE4 package in R (version 4.3.0) (https://www.R-project.org/). Alpha diversity indices were calculated using Mothur (v.1.30.2) [31] and box plots were generated using the BASE package in R. A network analysis was performed using the CoNet routine [32] in CYTOSCAPE (v.3.5) [33], and the top 50 nodes were visualized in GEPHI (v.0.10.1) [34]. Spearman correlation coefficients were calculated, and only those with a robust correlation (Spearman’s *r* > 0.5 or *r* < −0.5) and a statistically significant association (*p* < 0.05) were retained. The significance of different factors on community dissimilarity was tested with permutational multivariate analysis of variance (PERMANOVA) using the “adonis” function of the VEGAN package in R based on weighted UniFrac distances. A linear mixed model (LMM) was employed to identify the principal drivers of microbial alpha diversity. The phylogenetic tree was annotated and visualized using Interactive Tree of Life (iTOL) software (https://itol.embl.de/, accessed on 29 October 2024) [35]. A community bar plot analysis was conducted using the PANDAS package in Python (v.2.7) (http://www.python.org) based on the data sheets in the tax_summary_a folder. The relative abundance of taxa with a frequency of less than 1% was merged into the category “others”. A differential abundance analysis was conducted using the edgeR package in R [36]. The linear discriminant analysis (LDA) effect size (LEfSe) of the samples was based on different grouping conditions, including Wilcoxon’s *p* < 0.05 and a logarithmic LDA score > 2 [37]. The VEGAN package in R was employed for heat map analysis [38]. A Venn diagram analysis was performed using the BASE package in R [39]. The Mantel test was employed to investigate Spearman’s correlations between microbial communities, soil physicochemical and biotic characteristics, and soil enzyme activities using the VEGAN package in R. All statistical analyses were conducted in R. Nonparametric statistical tests (Kruskal–Wallis test or Wilcoxon test) were employed to evaluate alpha diversity and taxonomic differences among the different treatments.

## 3. Results

### 3.1. Ecological Restoration Significantly Enhanced Soil Microbial Diversity and Network Complexity Compared to Natural Grasslands

**Figure 2:**
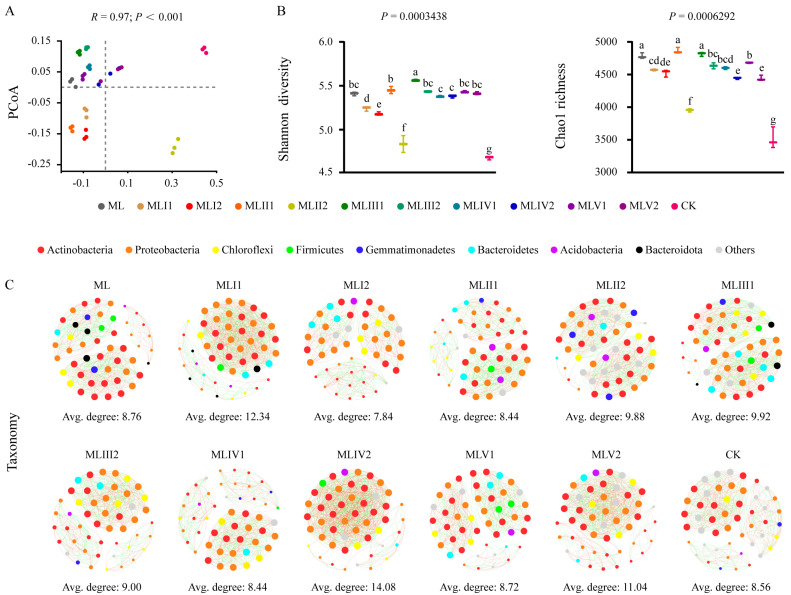
Ecological restoration has a strong effect on increasing microbial diversity and network complexity. (**A**) PCoA ordinations were conducted using Bray–Curtis distance matrices to analyze the microbial community structures from each treatment based on 36 samples (*n* = 36). (**B**) Microbial alpha diversity in different treatments. Different lowercase letters above the box plots indicate significant differences, as determined by the nonparametric Kruskal–Wallis test (*p* < 0.05). (**C**) Microbial co-occurrence networks in the different treatments. Avg. represents the average degree, indicating the degree of network complexity among the microorganisms. The size of each node represents its degree. The color of the line indicates a positive (green) or negative (red) correlation.

The overall microbial communities in the ecologically restored soils were significantly different from those in the control group (natural meadow), based on the PCoA ordination and nested PERMANOVA of the complete dataset (*R*^2^ = 27%, *p* < 0.001). Further analysis revealed that the microbial agent and fertilization (sheep manure and granular organic fertilizer) had significant effects on microbial community structure (Figure 2A; Table 1). The microbial agent exhibited an *R*^2^ value of 41.47% (*p* < 0.001), whereas fertilization exhibited an *R*^2^ value of 38.74% (*p* < 0.001).

To gain deeper insights into the effects of various ecological restoration methods on soil microbiomes, we evaluated the α diversity and co-occurrence patterns of the microbial communities. The results demonstrated that ecological restoration exerted a profound influence on microbial diversity, as evidenced by significant increases in both Shannon diversity and Chao1 richness than the control group did (Figure 2B). MLIII1 exhibited the highest Shannon diversity index, followed by MLII1, whereas MLII1 exhibited the highest Chao1 richness, followed by MLIII1 (Figure 2B). An LMM analysis of all samples further confirmed this trend, with *t*-values for Shannon diversity and Chao1 richness in the MLII1 group being 28.0 and 28.3, respectively, and in the MLIII1 group being 32.1 and 27.3, respectively, all higher than those in the other treatment groups. These findings strongly supported previous results (Table S3). However, from the perspective of species network complexity, these two restoration groups were not the highest (the average degrees for MLII1 and MLIII1 were 8.44 and 9.92, respectively) (Figure 2C; Table S4). This indicates that although ecological restoration may alter the structure of microbial communities, it does not necessarily alter the interaction patterns among microorganisms.

From a functional perspective, the results of the KEGG Orthology (KO) and COG analyses were consistent with those observed in Figure 2A,B (Figure S1A,B). However, in the functional network diagrams, the network complexities of MLII1 and MLIII1 were lower than those in the control group. Although the MLII2 group exhibited a lower Shannon diversity and Chao1 richness than the other treatment groups, its functional network complexity was highest (Figure S1C). This suggests that despite having fewer species, the microorganisms in this community play more crucial roles in executing key ecological functions or occupying more significant positions within the community.

### 3.2. Differential Assembly and Function of Soil Microbial Communities Following Ecological Restoration

**Figure 3:**
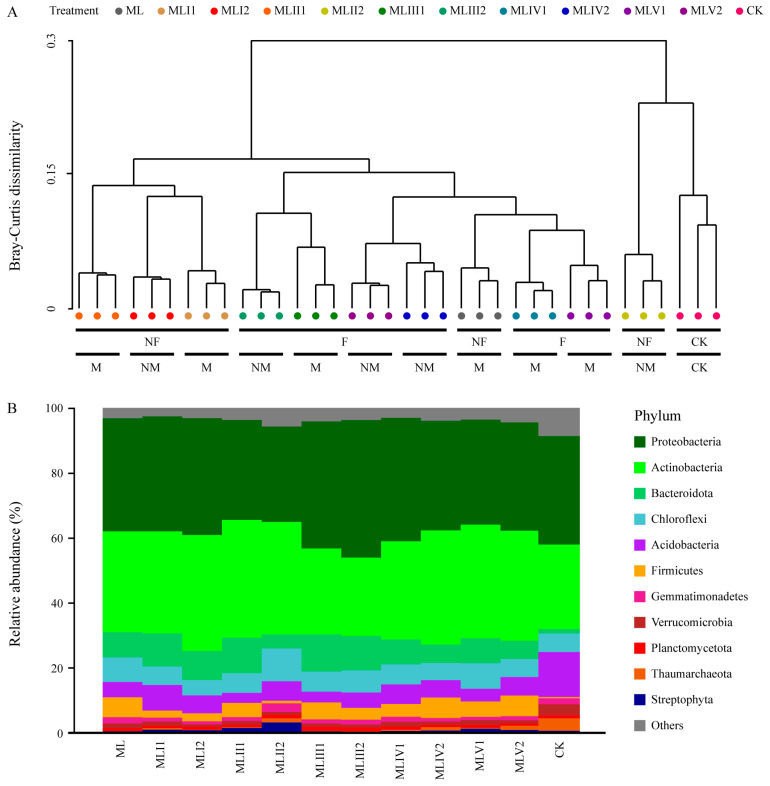
The microbial community structure and taxonomic composition varied among the different treatments. (**A**) Hierarchical clustering based on Bray–Curtis distances of the microbial phylum. NF, F, NM, and M represent no fertilization, fertilization, no microbial agent, and microbial agent, respectively. (**B**) Taxonomic composition of microbial communities in different treatments. Low-abundance phyla with less than 1% of the total sequence across all samples are grouped into “Others”.

**Figure 4:**
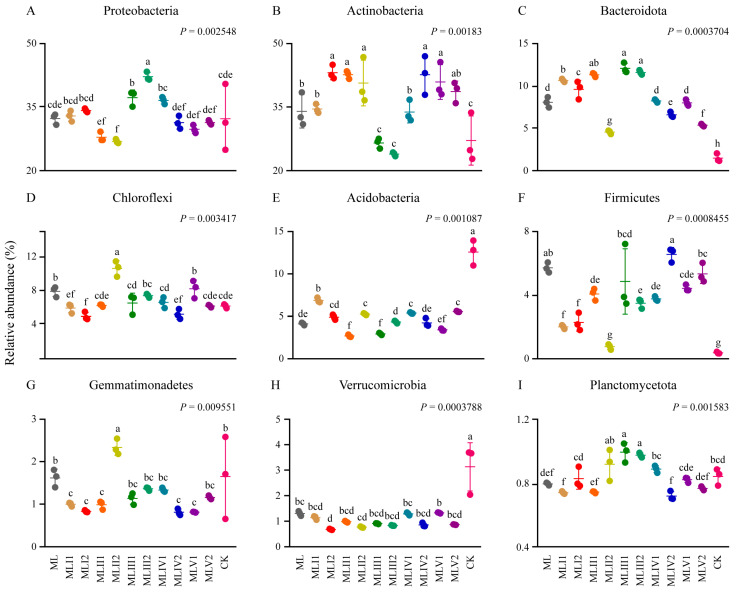
The relative abundances of the dominant phyla varied among the different treatments. (**A**–**I**) Relative abundance of dominant phyla in the different treatments. Different lowercase letters above the dots indicate significant differences (*p* < 0.05).

The hierarchical clustering analysis revealed that the microbial community in the MLII2 treatment group exhibited substantial similarity to that of natural meadow soil, forming a distinct cluster (Figure 3A). Furthermore, the MLI1, MLI2, and MLII1 treatment groups clustered together, as did the MLIII1, MLIII2, MLIV1, MLIV2, MLV1, and MLV2 treatment groups. This indicates that fertilization may exert a more pronounced influence on microbial community similarity than the microbial agent (Figure 3A). Across all treatment groups, Actinobacteria and Proteobacteria were the dominant phyla, whereas the relative abundances of Acidobacteria and Verrucomicrobia were significantly higher in the control group than in the treatment groups, and the relative abundance of Bacteroidetes was significantly lower in the control group (Figure 3B and Figure 4A–I).

The LEfSe analysis identified that *Luteimonas*, Deltaproteobacteria, Micrococcales, Actinomycetia, Chloroflexi, Gammaproteobacteria, Proteobacteria, Xanthomonadaceae, Firmicutes, Corynebacteriales, Bacilli, and Alphaproteobacteria were identified as the most significant biomarkers in the ML, MLI1, MLI2, MLII1, MLII2, MLIII1, MLIII2, MLIV1, MLIV2, MLV1, and MLV2 treatment groups and the CK control group (Figure S2A). A heatmap of the top 50 dominant functional groups indicated that the control group formed a distinct cluster (Figure S2B), which differed from the results shown in Figure 3. Consequently, further analysis was conducted to compare species composition and functionality between the MLII2 treatment and control groups.

Metabolic pathways and replication, recombination, and repair were identified as biomarkers in the MLII2 group, whereas a two-component system and inorganic ion transport and metabolism were identified as biomarkers in the control group (Figure S3A). The Venn diagram analysis revealed that 93.94% of the soil microbial phyla were shared between the MLII2 and the control groups. The two most prevalent phyla were Proteobacteria (32.99%) and Actinobacteria (31.78%). Cryptomycota was a distinctive feature of the MLII2 group, accounting for 49.72%, whereas the control group exhibited a higher proportion of uncultured microorganisms (Candidatus) (Figure S3B).

The results of the volcano plot analysis confirmed the microbial community differences between the MLII2 and control groups. The results indicated that Bacteroidetes, Fibrobacteres, and the three Candidatus phyla exhibited the most pronounced differences in relative abundance between the control and MLII2 groups despite their overall low abundance in the communities (Figure S3C). A Manhattan plot was used to compare the relative abundance of species with higher prevalence, focusing on the MLII2 and control groups. The relative abundance of Actinobacteria, Bacteroidetes, Chloroflexi, and Gemmatimonadetes was significantly higher in the MLII2 group than in the control group. In contrast, Acidobacteria and Verrucomicrobia exhibited significantly lower abundance. The relative abundance of Firmicutes, Planctomycetota, and Proteobacteria did not differ significantly (Figure S3D). A genus-level analysis also indicated that except for the Acidobacteria genera, which were less enriched in the MLII2 group, other phyla exhibited higher genus-level enrichment in the MLII2 group than in the control group (Figure S3D).

### 3.3. Soil Quality Assessment and the Correlation Between Microbial Communities and Environmental Factors

To further investigate the interactions between microbial communities and soil environments, we comprehensively analyzed biotic and abiotic indicators across treatment and control groups to assess soil quality. The results indicated that the soil quality in the treatment groups treated with fertilization and the microbial agent was generally superior to that in the untreated groups. However, the MLII2 treatment group exhibited the poorest soil quality compared to the other treatments (*F* = −2.4). OM content exhibited significant variation among the treatments, with the highest value observed in the MLIII1 group and the lowest in the MLII2 group. A similar pattern was observed for TN and TP, which reached their highest levels in MLIII1. This suggests that fertilization is effective in enhancing soil fertility. In contrast, AN and AK exhibited the most significant differences between the MLII2 and MLIII1 groups, reflecting their substantial impact on immediate nutrient availability for plants. Soil pH remained relatively stable across treatments, with minor variations that were unlikely to significantly affect soil biological properties (Table S5).

**Figure 5:**
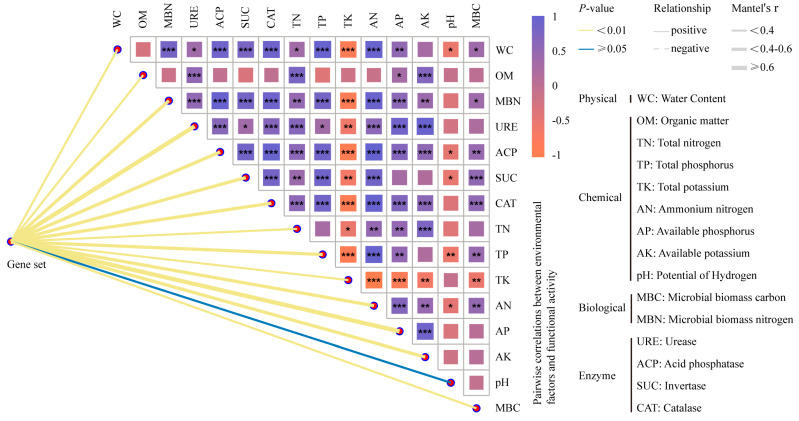
The potential drivers and ecological functions of microbial communities in the soil determined by Mantel tests were based on Spearman’s correlations. Correlations among microbial communities, soil physicochemical and biotic characteristics, and soil enzyme activity are shown. The edge width corresponds to the Mantel’s r statistic for the corresponding distance correlations (based on Bray–Curtis distance matrices of the microbial community and Euclidean distance matrices of environmental factor data), and the edge color denotes the statistical significance based on 999 permutations. Asterisks indicate the strength of Spearman’s rank correlation, with a greater number of asterisks indicating a higher correlation strength. The factors denoted in blue and orange suggest a substantial correlation between environmental factors and the microbial community.

The treatments significantly increased the MBC and MBN, particularly in the MLIII1 and MLII2 groups (Table S5). This indicates an enhanced microbial activity and biocatalytic potential. Mantel tests results demonstrated a significant positive correlation between microbial communities and all environmental factors except for pH (Figure 5; Table S6). TK and pH were negatively correlated with all other environmental factors (Figure 5).

Regarding chemical properties, AP exerted the most significant influence on microbial communities (*r* = 0.62), with a trend showing AP > AK > AN > OM. MBN exerted a greater influence on microbial communities than MBC. Regarding enzyme activities, URE exhibited the most significant impact (*r* = 0.76), followed by URE, SUC, CAT, and ACP (Table S6). These findings contribute to our understanding of the determinants of soil quality and provide a scientific basis for precise fertilization and microbial management strategies.

## 4. Discussion

The harsh environments at high altitudes render ecological conditions inherently fragile and susceptible to environmental disturbances. Therefore, successful restoration strategies must be implemented to maintain biodiversity and provide ecosystem services. Long-term coal mining activities in the Muli coalfield have resulted in significant environmental damage. Therefore, the objective of this study was to identify significant differences in microbial diversity and community composition resulting from various coal slag treatment measures. This was achieved through metagenomic and soil index measurements. In addition, the effectiveness of the different amelioration and restoration schemes was evaluated. This study is expected to provide essential theoretical support for the development of effective strategies to mitigate the environmental impacts of coal mining and improve degraded ecosystems.

### 4.1. Assemblies and Maintenance of Microbial Microbiomes

Microbially mediated soil biological processes are a crucial driving force in the restoration of terrestrial ecosystem functions. Conducting an integrated assessment of microbial diversity along with a microbial co-occurrence network analysis can illuminate the relationships between microorganisms, enabling a deeper understanding of the complex structures and interactions within microbial communities [40]. Overall, the results indicated that in the restoration group, there was an increase in soil microbial diversity, richness, and species network complexity compared to the control group (Figure 2B,C), which is consistent with the findings of Shu et al., who observed a rising trend in microbial diversity in restored ecosystems [41], indicating that these restoration efforts have begun to show results. This study also offered a detailed analysis of the impact of different coal slag treatments on microbial communities, a topic that has not been widely explored in the literature. The results showed that ecological restoration significantly increased the soil microbial diversity and richness, with the MLII1 and MLIII1 treatment groups performing exceptionally well (Figure 2; Table S3). This improvement could be attributed to the addition of microbial agents, attapulgite, sheep manure, and granular organic fertilizer. Previous studies have shown that *Lactobacillus* [42], *Bacillus subtilis* [43], and *Bacillus licheniformis* [44] are all considered rhizosphere-promoting bacteria. The addition of attapulgite improved the microenvironment of the compost, affecting microbial metabolism and thereby enhancing microbial activity [45,46]. Sheep manure [47] and organic fertilizer [48] not only increase soil organic matter and provide nutrients for crops but also promote the proliferation of soil microorganisms, thereby improving soil ecology, which is crucial for ecosystem resilience [49]. In general, microbial communities with more complex networks are better for more efficient material and energy cycling in ecosystems compared to those with simpler networks. This aids in maintaining ecosystem functions and provides greater resistance to environmental stress [50]. However, in terms of functional network complexity, these groups were actually lower than that of the control group (Figure S1C). It is worth noting that the MLII2 group exhibited a lower Shannon diversity index but was the only group with a higher functional network complexity that surpassed that of the control group (Figure S1C). This finding conflicts with that of Guerrero-Preston et al. [51], who reported a positive correlation between taxonomic and functional diversity. Our findings indicate that certain microbial communities can sustain high functional complexity despite lower taxonomic diversity, enhancing ecosystem stability and resilience under specific conditions or due to the presence of keystone species [11]. Consequently, MLII2 treatment, which exhibits functional diversity advantages, may outperform other treatments in ecological restoration and ecosystem service enhancement, and this treatment is likely to promote the rapid recovery of ecosystem functions and improve soil quality. Meanwhile, it is important to note that acidic metal salts, such as iron, which is a significant redox-active transition metal, play a crucial role in the carbon cycle of ecosystems [52]. However, iron toxicity often causes severe disruptions in plant morphology and physiology. These disruptions can include reduced germination rates, interference with enzyme activity, nutrient imbalances, membrane damage, and alterations in the chloroplast ultrastructure [53]. Therefore, long-term monitoring is essential for a comprehensive evaluation.

### 4.2. The Keystone Microbial Taxa and Their Ecological Functions

The integration of the microbial agent into the restoration strategies of the Muli coal mine introduced a range of microorganisms that were potentially absent in the native soils. This introduction is likely to have altered the existing microbial community structure and function, as evidenced by the changes in the taxonomic composition and functional profiles of the soil microbiomes (Figure 3, Figure 4 and Figure S2). This indicates that certain species may be capable of colonizing and proliferating in restored environments, potentially outcompeting native species and altering ecosystem processes. Actinomycetes are Gram-positive bacteria and one of the oldest and most promising bio-exploration resources, widely distributed in soil, aquatic environments, plant litter, compost, and food, as well as extreme environments; they play a crucial ecological role in carbon and nutrient cycling, demonstrate strong adaptability to environmental changes, and are extensively used in steroid transformation, petroleum dewaxing, and wastewater treatment, making them a dominant component of the soil microbial community [54,55,56]. Proteobacteria are typically fast-growing highly adaptable symbiotic bacteria that follow a K-strategy lifestyle [57,58]. Therefore, the transfer of these key functional groups may enhance soil fertility and aid the recovery of plant communities.

The high-altitude meadow area under study may act as an environmental filter, selecting for microorganisms with broad physiological traits that enable them to adapt to the environmental conditions and maintain a certain level of richness, diversity, and functionality in the face of environmental changes [59,60]. The relative abundances of Acidobacteria and Verrucomicrobia were higher in natural meadow soils than in all restoration groups (Figure 4). Previous studies have indicated that Acidobacteria thrive under diverse environmental conditions, including extreme acidity and nutrient-poor environments. These bacteria play pivotal roles in OM decomposition and nutrient cycling. For example, some Acidobacteria can utilize low-molecular-weight organic compounds as energy and carbon sources, enabling them to survive and function under nutrient-poor conditions [61,62]. Although Verrucomicrobia are less abundant in natural environments, their widespread distribution suggests that they can adapt to various environmental conditions [63]. In soil ecosystems, Verrucomicrobia may participate in the decomposition of OM, particularly during the nitrogen and carbon cycles. Despite the limited number of studies on their role in cold nutrient-poor plateau environments, their metabolic diversity and ecological functions suggest that these microbes can adapt to such conditions. Given the harsh conditions and nutrient-poor soils in high-altitude regions, this study postulated that the high relative abundance of Acidobacteria and Verrucomicrobia in natural meadow soils reflects their importance in maintaining soil OM decomposition and nutrient cycling, which supports soil productivity and ecosystem function under extreme conditions. Furthermore, their high abundance may be attributed to their distinctive ecological niches in high-altitude regions. For instance, they may engage in specific plant–microbe interactions or help withstand common environmental stresses in high-altitude areas, such as low temperatures, UV radiation, and aridity [64].

Collectively, these findings suggest that in high-altitude ecological restoration and management practices, the ecological functions and potential value of Acidobacteria and Verrucomicrobia should be fully considered. Further investigation is required to elucidate their precise roles in high-altitude ecosystems and determine the most effective means of harnessing their activities to enhance ecosystem health and stability. This study provides valuable insights into the drivers of microbial diversity and function in plateau ecosystems through a detailed analysis of microbial communities during the ecological restoration of the Muli mining area.

### 4.3. Microbial Interactions with Soil Environment

The intricate interactions between soil microbial communities and environmental factors underscore the multifaceted nature of ecological restoration and highlight the importance of environmental factors in shaping microbial assemblages [65]. The Mantel test revealed a negative correlation between TK and other environmental factors (Figure 5), suggesting that K levels may be influenced by a range of biotic and abiotic variables. These include the soil pH, OM content, and microbial activity. A strong positive correlation between AP and microbial community differences (*r* = 0.62) (Figure 5; Table S6) highlights the pivotal role of nutrient availability in shaping the microbial community structure [66]. Previous studies have demonstrated that AP enhances microbial metabolism and enzyme activity, thereby increasing OM decomposition and nutrient cycling [67]. Given the harsh environmental conditions at high altitudes, characterized by low temperatures and short growing seasons, these factors could limit nutrient cycling. Consequently, the findings presented are of paramount importance for the maintenance of ecosystem balance in these distinctive and vulnerable systems that are adapted to cold and nutrient-poor conditions.

pH was the only soil chemical parameter that exhibited a negative correlation with microbial communities, although this was not statistically significant in this study (Figure 5; Table S6). pH has a profound influence on microbial physiological activities and ecological functions. Minor pH variations among treatments can significantly affect microbial survival and activity because many microbes have specific pH preferences [68]. It is of paramount importance to maintain a stable and optimal pH range to foster diverse and functionally rich microbial communities that support the overall health and resilience of ecosystems.

Our study demonstrated that biological factors, including MBC and MBN, significantly influenced microbial community composition (Table S6). This finding corroborates the hypothesis proposed by Robinson et al. [36] that soil microbial biomass is a pivotal determinant of soil health and function. However, compared to the control group, the majority of the restoration groups exhibited a lower MBC and MBN, which may be attributed to the incomplete recovery of microbial communities during the early stages of restoration. These findings indicate that microbial diversity and biomass must be considered holistically to enhance the overall soil health and functionality during ecological restoration.

Understanding the interactions between soil microbial communities and environmental factors is of paramount importance for the development of effective restoration strategies that optimize nutrient utilization and promote soil ecosystem balance and productivity. Therefore, it is imperative to assess the long-term consequences of contemporary ecological restoration strategies. Although this study demonstrated the immediate benefits of these treatments, the persistence and stability of these effects over time remains to be fully evaluated. This is a limitation of this study. As previously noted by Northey et al. [4], long-term monitoring is essential for assessing the lasting effects of ecological restoration and for guiding future management decisions.

This study offers valuable insights into the intricate interactions between soil microbial communities and environmental factors during the ecological restoration of high-altitude coal mining areas. Significant improvements in microbial diversity and function in Muli coal mine soils are crucial for the long-term health and stability of ecosystems. These findings contribute to our understanding of the role of soil microbes in ecosystem restoration and provide a foundation for the development of more effective restoration strategies in similar environments.

## 5. Conclusions

This study evaluated ecological restoration measures in the Muli coal mine area of Qinghai Province. This revealed that the MLII2 treatment group was highly effective in restoring microbial community structure and function. Although MLII2 exhibited lower species diversity than the other treatment groups, its high similarity to natural meadow soil microbial communities and high functional network complexity indicate its unique advantages in ecological restoration. This finding underscores the importance of not only enhancing microbial diversity but also prioritizing the restoration of community functions, particularly for the recovery of fragile high-altitude ecosystems. Consequently, the MLII2 treatment strategy, which demonstrated comprehensive effectiveness in restoring soil microbial community structure and function, is regarded as the most reasonable ecological restoration method. The findings of this study offer valuable scientific support and a theoretical foundation for ecological restoration, providing an actionable framework and practical guidance for effective soil remediation, biodiversity enhancement, and ecological protection in this region and similar high-altitude mining areas.

## Figures and Tables

**Table 1:** Effects of ecological restoration, microbial agents, and fertilization on the microbial community based on PERMANOVA.

Group	*F* Value	*R*^2^ (%)	Pr (>*F*)
G1	33.60	93.90	<0.001
G2	12.58	27.00	<0.001
G3	11.69	41.47	<0.001
G4	10.43	38.74	<0.001

G1 represents a comprehensive PERMANOVA encompassing 11 treatment groups and a control group. The objective of this analysis was to quantitatively identify the differences among the microbial communities. G2 provided a baseline assessment of treatment effects by comparing the overall microbial community structures of the 11 treatment groups with those of the control group. G3 was designed to investigate the differences in microbial community responses to treatments with and without the microbial agent. G4 further investigated the sensitivity of microbial communities to fertilization treatments (sheep manure and granular organic fertilizer) by comparing them with the unfertilized groups.

## Data Availability

Data presented in this study can be found in the NCBI for Biotechnology Information SRA repository (accession number: SRP505773).

## Supplementary Materials

The following supporting information can be downloaded at: https://www.mdpi.com/article/10.3390/microorganisms12112222/s1.
