# Supplementary material for: Metagenomic Insights into Coal Slag Remediation Effects on Soil and Microbial Health in Qinghai’s Muli Coal Mine
Source: Microorganisms. 2024 Nov 1;12(11):2222. doi: 10.3390/microorganisms12112222 (PMC11596568; doi:10.3390/microorganisms12112222)
Supplement: Supplementary file 1 [file microorganisms-12-02222-s001.zip › microorganisms-3273894-supplementary.pdf]

Metagenomic Insights into Coal Slag Remediation Effects on Soil and Microbial Health in Qinghai's Muli Coal Mine

Qi Lin [1], Pan Yang [1], Yongbei Zhang [2], Wenfei Zhang [1,*] and Hongping Wu [1,*]

[1] Ministry of Education Key Laboratory for Ecology of Tropical Islands, College of Life Sciences, Hainan Normal University, Haikou 571158, China; 20202071300170@hainnu.edu.cn (Q.L.); y486269195@gmail.com (P.Y.)

[2] Hainan Beiou Bio-Energy Development Co., Ltd., Haikou 570100, China; zybei2013@yeah.net

* Correspondence: wfzhang@hainnu.edu.cn (W.Z.); whp789@126.com (H.W.)

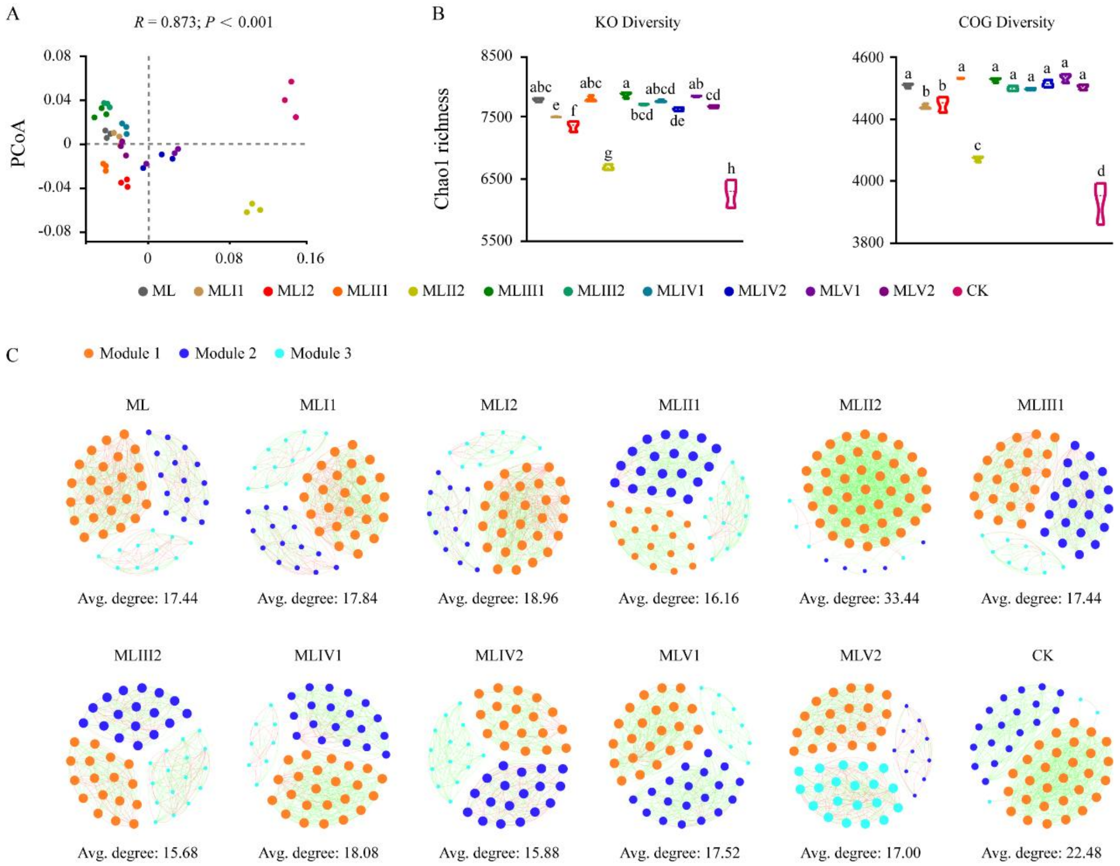

Figure S1. Functional profiles of soil microbiomes. A: PCoA ordinations were conducted using Bray–Curtis distance matrices of KO, revealing that the treatment microbiome differed significantly from the control microbiome (n = 36). B: Violin Plot showing the functional diversity (including KO and COG) of the treated and control microbiomes. Different lowercase letters above the violins indicate a significant difference as determined by the nonparametric Kruskal–Wallis test ($P < 0.05$). C: Microbial functional network for each treatment.

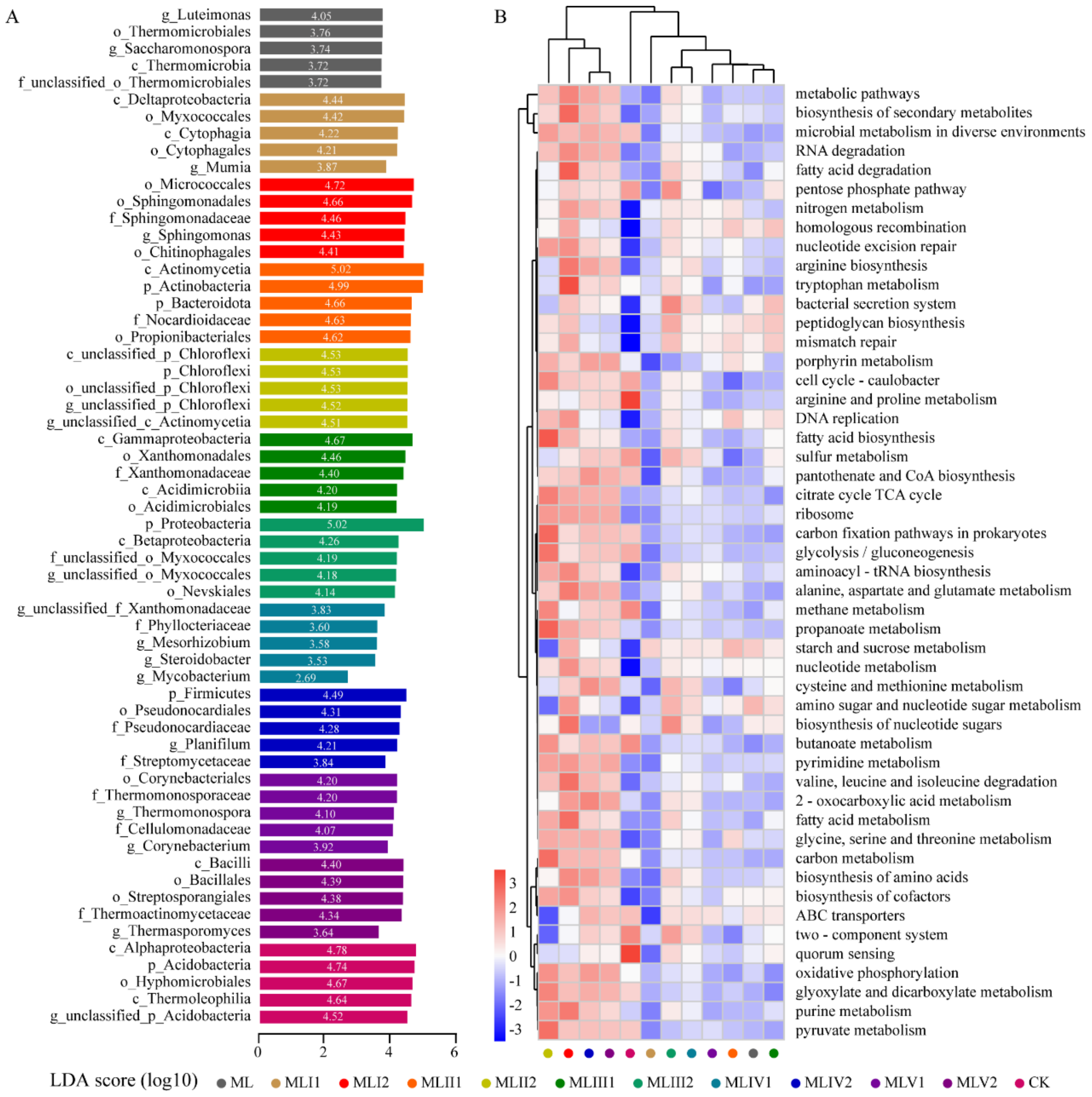

Figure S2. The biomarker taxa and potential functional groups of the microbial communities varied among treatments. A: LEfSe identifying biomarker taxa associated with different treatments. Only the five most specific biomarker taxa are shown. B: Heatmap showing the relative abundance of the dominant functional groups (top 50).

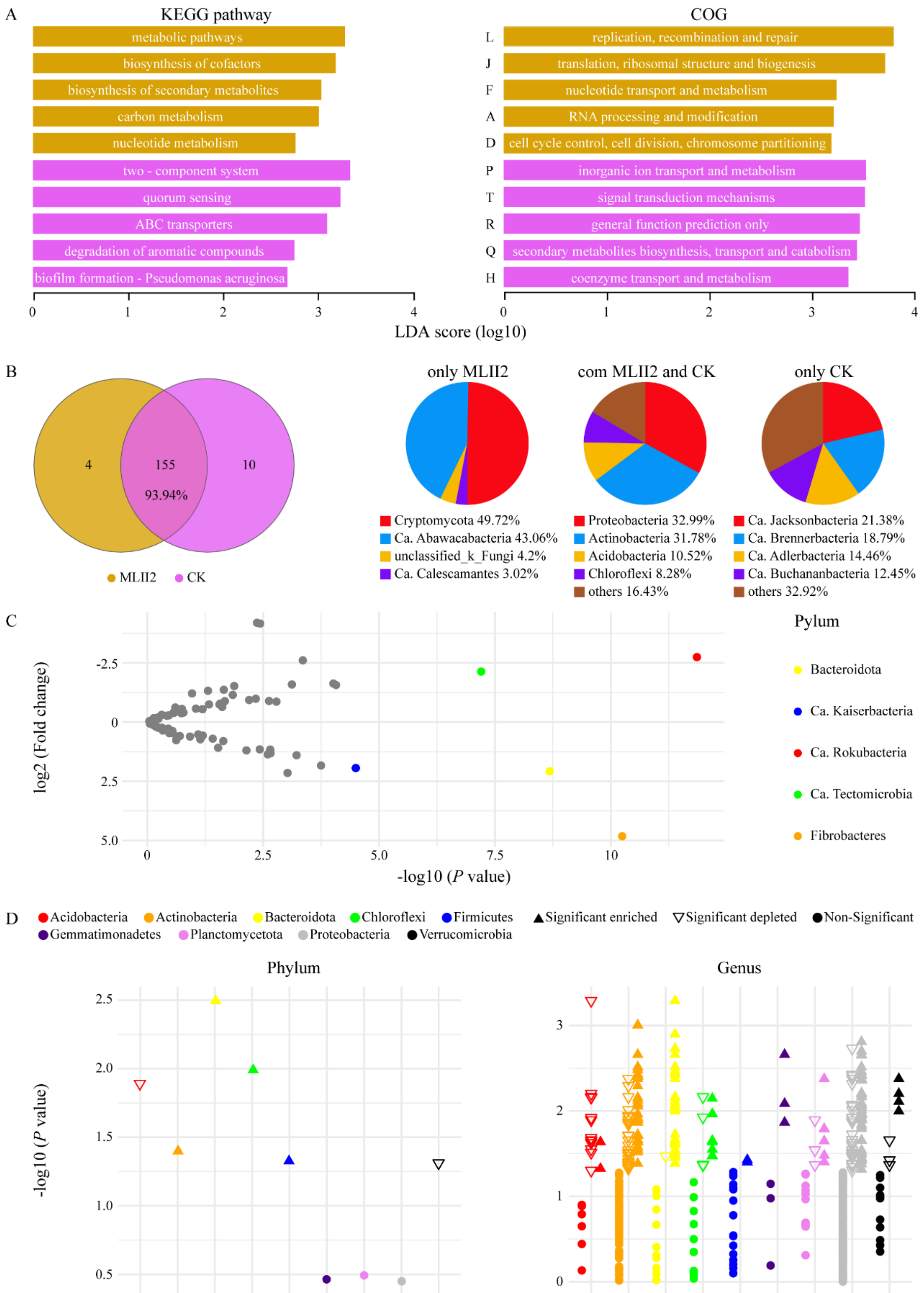

Figure S3. Specific microbial taxa and functions in MLII2 and CK. A: LEfSe identifying biomarker functions for MLII2 and CK. Only the five most specific biomarkers are presented. B: Venn plot showing shared and specific phyla between MLII2 and CK. C: Volcano plot illustrating the enrichment and depletion patterns of microbial communities in CK compared to those in MLII2. Each point represents a single phylum. Each colored point above the x-axis at zero represents an individual depleted phylum, and each colored point below the x-axis at zero represents an individual

enriched phylum. The position along the y-axis represents the fold change in abundance compared with MLII2, and the x-axis reports the *P* values. Ca. represents Candidatus. D: Manhattan plots showing phylum enrichment or depletion of MLII2 in soil microbiota. Each circle or triangle represents a single phylum or genus. Phylum/genera enriched or depleted in MLII2 are represented by filled or empty triangles, respectively (genus abundance > 0.01%, $P < 0.05$).

Table S1. Detailed on soil treatment methods and their compositions.

Treatments	Code	Compositions
1	CK	Natural soil from Qinghai's Muli Coal Mine meadows
2	ML	Microbial agent[a]
3	MLI1	Microbial agent[a], Indigenous microbial agent[b]
4	MLI2	Indigenous microbial agent[b]
5	MLII1	Microbial agent[a], Chemical weathering agent[c], Attapulgite[d]
6	MLII2	Chemical weathering agent[c], Attapulgite[d]
7	MLIII1	Microbial agent[a], Sheep manure (2.4 kg/m²), Granular organic fertilizer (1.2 kg/m²)
8	MLIII2	Sheep manure (2.4 kg/m²), Granular organic fertilizer (1.2 kg/m²)
9	MLIV1	Microbial agent[a], Sheep manure (3.6 kg/m²), Granular organic fertilizer (1.2 kg/m²)
10	MLIV2	Sheep manure (3.6 kg/m²), Granular organic fertilizer (1.2 kg/m²)
11	MLV1	Microbial agent[a], Sheep manure (4.8 kg/m²), Granular organic fertilizer (0.8 kg/m²)
12	MLV2	Sheep manure (4.8 kg/m²), Granular organic fertilizer (0.8 kg/m²)

[a]Microbial agent contains *Lactobacillus* sp., *Bacillus subtilis*, and *Bacillus licheniformis*, and was applied to the sample site with bacterial content exceeding 2×10^{12} CFU/g.

[b]Indigenous microbial agent includes Acidobacteria and Verrucomicrobia species, *Bradyrhizobium erythrophlei*, *Cadophora* sp., and *Rhizophagus irregularis*.

[c]Weathering agent includes acidic metal salts, such as calcium and iron.

[d]Attapulgite powder has a porous structure with water retention and slow-release fertilizer properties.

Table S2. Overview of shotgun metagenomic data.

Soil microorganism metagenome		
Quality control	Raw reads	1,587,167,466
	QC reads	1,518,468,654
	Microbial reads	539,727,640
Assembly	Contigs	12,442,006
	Total length	6,992,678,904
	Mean length	194,241,080
Annotations	Predicted genes	11,595,869
	Assigned to NCBI NR database (%)	76.20%
	Assigned to KEGG database (%)	40.42%
	Assigned to COG database (%)	29.86%

Microbial reads: plant sequence elimination and selective annotation of bacteria and fungi.

Table S3. Effects of different treatments on microbial alpha diversity based on linear mixed models (LMM).

Treatment	Shannon diversity		Chao1 richness	
	t value	Pr (>*t*)	*t* value	Pr (>*t*)
ML	26.5	<2e-16	26.7	<2e-16
MLI1	20.4	<2e-16	22.2	<2e-16
MLI2	18.3	<2e-16	21.2	<2e-16
MLII1	28.0	1.40e-15	28.3	<2e-16
MLII2	5.8	<2e-16	9.2	2.29e-9
MLIII1	32.1	5.83e-6	27.3	<2e-16
MLIII2	27.4	<2e-16	23.5	<2e-16
MLIV1	25.4	<2e-16	22.8	<2e-16
MLIV2	25.4	<2e-16	19.5	3.25e-16
MLV1	27.3	<2e-16	24.5	<2e-16
MLV2	26.7	<2e-16	19.5	3.31e-16

Table S4. Microbial co-occurrence network characteristics for each treatment.

Treatment	Node	Positive edge	Negative edge	Average degree	Modularity
ML	50	231	207	8.76	0.563
MLI1	50	305	312	12.34	0.169
MLI2	50	214	178	7.84	0.666
MLII1	50	266	156	8.44	0.580
MLII2	50	256	238	9.88	0.519
MLIII1	50	284	212	9.92	0.511
MLIII2	50	235	215	9.00	0.491
MLIV1	50	230	192	8.44	0.560
MLIV2	50	343	361	14.08	0.103
MLV1	50	238	198	8.72	0.571
MLV2	50	270	282	11.04	0.275
CK	50	208	220	8.56	0.555

Table S5. Soil properties and quality assessment.

Treatment	WC	OM	TN	TP	TK	AN	AP	AK	pH	MBC	MBN	URE	ACP	SUC	CAT	F
ML	10.27 ± 0.04 i	223.67 ± 2.04 e	3.05 ± 0.11 f	0.60 ± 0.01 g	15.04 ± 0.07 j	92.90 ± 0.2 g	39.81 ± 0.14 c	308.00 ± 1.00 e	8.76 ± 0.01 b	67.52 ± 0.64 j	36.27 ± 0.72 g	3.83 ± 0.06 f	3.73 ± 0.04 f	6.34 ± 0.07 j	5.69 ± 0.19 h	-0.7
MLI1	8.77 ± 0.05 k	257.75 ± 0.94 c	3.79 ± 0.04 e	0.45 ± 0.00 j	18.07 ± 0.18 d	68.17 ± 0.44 i	9.35 ± 0.05 h	356.00 ± 1.00 d	9.18 ± 0.04 a	333.05 ± 0.07 e	32.61 ± 0.23 h	5.88 ± 0.03 d	3.99 ± 0.03 e	26.22 ± 0.22 g	6.11 ± 0.02 g	-0.69
MLI2	7.21 ± 0.11 l	360.48 ± 0.52 a	5.53 ± 0.11 c	0.45 ± 0.01 j	22.15 ± 0.07 a	31.90 ± 0.96 l	2.52 ± 0.04 j	255.00 ± 3.00 g	8.47 ± 0.02 de	140.78 ± 0.45 h	18.25 ± 0.25 k	2.56 ± 0.03 i	1.00 ± 0.04 i	6.66 ± 0.05 j	3.90 ± 0.12 j	-1.53
MLII1	9.41 ± 0.14 j	169.70 ± 0.76 g	2.59 ± 0.08 g	0.57 ± 0.01 h	19.04 ± 0.08 c	59.59 ± 0.84 j	25.93 ± 0.09 f	278.00 ± 2.00 f	7.98 ± 0.03 i	124.74 ± 0.74 i	28.54 ± 0.13 j	3.05 ± 0.04 g	3.57 ± 0.09 g	21.68 ± 0.18 i	5.02 ± 0.01 i	-0.79
MLII2	12.10 ± 0.02 h	88.12 ± 0.14 i	1.53 ± 0.11 i	0.48 ± 0.01 i	19.50 ± 0.06 b	34.42 ± 0.59 k	0.70 ± 0.02 k	95.33 ± 0.58 j	8.47 ± 0.02 de	43.96 ± 0.26 k	16.22 ± 0.31 l	0.35 ± 0.04 l	1.64 ± 0.05 h	4.15 ± 0.01 k	2.72 ± 0.02 k	-2.4
MLIII1	72.62 ± 0.07 a	309.68 ± 0.99 b	10.06 ± 0.34 a	1.23 ± 0.01 a	13.66 ± 0.12 k	429.80 ± 2.79 a	153.52 ± 0.60 a	1287 ± 6.66 a	8.23 ± 0.03 g	1334.94 ± 0.31 a	44.53 ± 0.48 e	9.59 ± 0.02 a	12.67 ± 0.13 b	65.58 ± 0.38 c	10.36 ± 0.09 a	4.95
MLIII2	63.78 ± 0.04 b	175.61 ± 1.30 f	7.82 ± 0.11 b	0.99 ± 0.02 c	16.30 ± 0.21 g	389.65 ± 2.23 b	111.47 ± 0.19 b	384.00 ± 1.00 b	8.16 ± 0.04 h	22.41 ± 0.23 l	69.92 ± 0.34 a	7.81 ± 0.03 b	12.80 ± 0.07 a	71.79 ± 0.32 b	10.10 ± 0.02 b	2.36
MLIV1	20.08 ± 0.04 e	99.12 ± 0.32 h	3.29 ± 0.17 f	0.76 ± 0.01 d	17.81 ± 0.14 e	165.13 ± 0.65 d	26.83 ± 0.08 e	278.67 ± 0.58 f	8.46 ± 0.01 e	649.50 ± 0.65 c	52.77 ± 0.30 c	6.00 ± 0.02 c	6.33 ± 0.14 d	49.69 ± 0.39 d	8.20 ± 0.03 d	0.16
MLIV2	15.34 ± 0.02 f	59.74 ± 1.31 k	2.25 ± 0.07 h	0.70 ± 0.01 e	17.50 ± 0.13 f	119.32 ± 1.17 f	15.30 ± 0.02 g	231.00 ± 1.00 h	8.51 ± 0.01 d	321.98 ± 0.70 f	38.50 ± 0.58 f	2.94 ± 0.01 h	4.07 ± 0.04 e	33.39 ± 0.22 e	7.12 ± 0.04 f	-0.9
MLV1	32.24 ± 0.05 d	252.32 ± 0.68 d	4.36 ± 0.21 d	0.64 ± 0.01 f	15.99 ± 0.12 h	130.99 ± 0.15 e	29.03 ± 0.08 d	371.33 ± 2.08 c	8.60 ± 0.02 c	526.70 ± 0.68 d	48.56 ± 0.60 d	4.90 ± 0.01 e	6.25 ± 0.08 d	24.89 ± 0.29 h	8.63 ± 0.08 c	0.75
MLV2	13.04 ± 0.06 g	57.32 ± 0.76 l	1.79 ± 0.16 i	0.65 ± 0.01 f	18.18 ± 0.27 d	90.70 ± 0.62 h	7.40 ± 0.02 i	115.33 ± 0.58 i	8.74 ± 0.01 b	165.38 ± 1.11 g	29.44 ± 0.74 i	2.07 ± 0.05 j	3.55 ± 0.08 g	28.49 ± 0.07 f	6.22 ± 0.04 g	-1.51
CK	52.42 ± 0.04 c	76.18 ± 0.71 j	3.79 ± 0.14 e	1.13 ± 0.01 b	15.47 ± 0.11 i	215.45 ± 0.68 c	2.61 ± 0.07 j	98.67 ± 2.52 j	8.36 ± 0.01 f	669.06 ± 0.43 b	57.79 ± 0.59 b	0.93 ± 0.05 k	7.67 ± 0.07 c	125.94 ± 0.20 a	7.34 ± 0.10 e	0.3

Data represent the mean ± SE. Different lowercase letters above the dots indicate significant differences ($P < 0.05$).

Table S6. Effects of multiple biotic and abiotic factors on the microbiome assembly.

Classification	Env factors	*r*	*P*
Physical	WC	0.37	0.001
Chemical	OM	0.33	0.001
	TN	0.30	0.004
	TP	0.51	0.001
	TK	0.29	0.001
	AN	0.48	0.001
	AP	0.62	0.001
	AK	0.53	0.001
	pH	0.07	0.208
Biological	MBC	0.21	0.008
	MBN	0.44	0.001
Enzyme	URE	0.76	0.001
	ACP	0.45	0.001
	SUC	0.50	0.001
	CAT	0.48	0.001

The significance of the different factors on microbial community dissimilarity was tested using PERMANOVA (based on weighted UniFrac distances). Env denotes the environment.
